# Evaluation of optical and electronic properties of silicon nano-agglomerates embedded in SRO: applying density functional theory

**DOI:** 10.1186/1556-276X-9-507

**Published:** 2014-09-17

**Authors:** Néstor D Espinosa-Torres, David Hernández-de la Luz, José Francisco J Flores-Gracia, José A Luna-López, Javier Martínez-Juárez, Diana E Vázquez-Valerdi

**Affiliations:** 1 Instituto de Ciencias, Centro de Investigaciones en Dispositivos Semiconductores, Benemérita Universidad Autónoma de Puebla, C.U. Edif. 103 C-D, Col. San Manuel, Puebla Pue., C.P. 72570, Mexico

**Keywords:** Nano-agglomerates, Silicon clusters, Silicon rich oxide, Luminescence, HRTEM, DFT

## Abstract

In systems in atomic scale and nanoscale such as clusters or agglomerates constituted by particles from a few to less than 100 atoms, quantum confinement effects are very important. Their optical and electronic properties are often dependent on the size of the systems and the way in which the atoms in these clusters are bonded. Generally, these nanostructures display optical and electronic properties significantly different to those found in corresponding bulk materials. Silicon agglomerates embedded in silicon rich oxide (SRO) films have optical properties, which have been reported to be directly dependent on silicon nanocrystal size. Furthermore, the room temperature photoluminescence (PL) of SRO has repeatedly generated a huge interest due to its possible applications in optoelectronic devices. However, a plausible emission mechanism has not been widely accepted in the scientific community. In this work, we present a short review about the experimental results on silicon nanoclusters in SRO considering different techniques of growth. We focus mainly on their size, Raman spectra, and photoluminescence spectra. With this as background, we employed the density functional theory with a functional B3LYP and a basis set 6-31G* to calculate the optical and electronic properties of clusters of silicon (constituted by 15 to 20 silicon atoms). With the theoretical calculation of the structural and optical properties of silicon clusters, it is possible to evaluate the contribution of silicon agglomerates in the luminescent emission mechanism, experimentally found in thin SRO films.

## Background

Small silicon clusters can be obtained, for instance, when using photochemical etching treatment and pulsed laser evaporation method which in turn show also photoluminescence (PL). Nevertheless, such small silicon clusters are short-lived intermediate species, and it is very difficult to control the number of atoms in the Si clusters formed. On the other hand, some silicon clusters have been prepared by organic synthesis methods, and a similarity of the PL and absorption spectra to those measured in porous Si (p-Si) has been pointed out. With respect to the latter material, it is currently agreed that the quantum confinement effects and the hydrogen saturation (surface effects) of Si nanocrystallites play key roles in the origin and mechanism of PL in p-Si where strong visible PL is observed when it is fabricated by electrochemical anodization [1]. Besides, their optical and electronic properties have caught much attention from the perspective of solid-state physics and its application to optical devices [2,3].

Kanemitsu and co-workers [4,5] and Furukawa et al. [6] synthesized several kinds of Si clusters, Si backbone polymers, network polymers, and planar siloxane structures and studied their optical properties to understand the dimensional effects of Si-based nanostructures such as p-Si. In the chain clusters and polymers, sharp PL bands were observed with very small Stokes shifts. On the other hand, following the line of nanostructures with Si clusters, we find that such structures which have generated great interest is the silicon rich oxide (SRO) thin film; this material exhibits optical properties in the same manner to p-Si, but it is significantly less chemically reactive than p-Si.

Si-nanocrystals (Si-nCs) embedded in dielectric matrices such as silicon dioxide exhibit unique optical and electrical properties which are determined by quantum size and Coulomb blockade effects [2]. Si-nCs can emit and absorb light at energies which can be controlled by their sizes, i.e., their gaps can be tuned. This latter fundamental property of Si-nCs is very useful in third-generation solar cells [7].

Commonly, SRO is considered as a multi-phase material constituted by a mixture of silica (SiO_2_), off-stoichiometric oxides (SiO_*x*_, *x* < 2), and elemental silicon. After thermal treatment at temperatures above 1,000°C, the off-stoichiometric oxides, SiO_*x*_ (*x* < 2), react to produce silicon nanoclusters, structures with different oxidation states with or without defects, and silica [8]. Silicon nanocrystals and silicon agglomerates have been characterized in SRO films employing transmission electron microscopy (TEM), high-resolution transmission electron microscopy (HRTEM), energy-filtered transmission electron microscopy (EFTEM), X-ray diffraction (XRD), and atomic force microscopy (AFM) [9] as characterization techniques. The formation, shape, and size of Si-nCs depend on the excess silicon and annealing parameters (time and temperature) and also on the type of carried gas used to grow nanostructures.

According to Raghavachari and Rohlfing [10], the behavior of small-sized silicon clusters is frequently correlated with the trend of binding energy per atom as a function of cluster size. In this type of systems, the electronic configuration corresponding to both a single atom and a complex of atoms in the cluster is a determining factor in the cluster stability [11].

Over the past 20 years, medium-sized silicon clusters Si_*n*_ (*n* > 10) have attracted much attention both experimentally [12] and theoretically [13]. Considerable effort has been devoted to determine the ground-state geometric structures, namely the global minima as a function of the cluster size *n*. For *n* ≤ 7, the global minima are firmly established by both *ab initio* calculations and Raman/infrared spectroscopy measurements, whereas for *n* ≤ 12 the global minima based only on *ab initio* calculations [14-16] are well accepted.

For 13 ≤ *n* ≤ 20, an unbiased search for the global minima has been undertaken based on either the genetic algorithm coupled with semi-empirical tight-binding (TB) technique [17] or the single-parent evolution algorithm coupled with density functional (DF), TB, and density functional theory (DFT) methods [18,19]. Establishing successfully the geometry corresponding to the global minimum energy is a critical step for a further reliable evaluation of the optical and structural properties, and thereby it contributes properly to the understanding of the underlying mechanisms of luminescence.

It is well-known that crystalline silicon (c-Si) has an indirect band gap, which means that every optical transition must be accompanied by the creation or annihilation of a phonon. Besides, this material presents an optical disadvantage attributed to low band gap value *E*_g,c-Si_ = 1.12 eV at room temperature (RT), corresponding to a wavelength *λ*_g,c-Si_ = 1,107 nm. This fact leads to the radiation emitted by, for example, a light-emitting diode (LED) made of c-Si which corresponds to the infrared emission and then it is non-visible by the human eye.

However, by using nanoscaled silicon structures, the optical disadvantage can be overcome, because of the presence of radiative optical transitions brought about in quantum confined states of Si nanostructures which generate visible radiation, but the disadvantage of the indirect band gap still remains. Average PL decay times *τ*_PL_ for Si-nCs with diameters *d* ~ 3.4 nm are reported to be in the order of 100 to 500 μs at RT [20].

Broadly, the SRO can be obtained by different techniques; however, the low-pressure chemical vapor deposition (LPCVD) technique is frequently used because it results a simple method that easily allows the controlled fluctuations of silicon excess. In this technique, the partial pressure ratio caused by the flow of reactive gases, defined as Ro = *P*(N_2_O)/*P*(SiH_4_), is used to determine the silicon excess. For example, for Ro = 3, we have a silicon excess of 17 at.%, and Ro = 100 is used to obtain stoichiometric silicon dioxide. When SRO films are obtained by LPCVD technique, the most intense light emission observed corresponds to films with approximately 5 at.% silicon excess as reported, although silicon nanocrystals were not observed in such films [21].

It is possible that small-sized silicon agglomerates (Si_*n*_, *n* < 20) were present in these particular films (for the case of Ro = 30) which would hardly be detected due to atomic instead of nanoscale dimensions. Size regimes in the evolution of semiconductor spectroscopic properties were introduced by Efros and Efros [22]. For many years, different methods have been used for the preparation of silicon nanocrystals, e.g., chemical vapor deposition [23], Si ion implantation [24], colloidal synthesis [25], magnetron sputtering [26], and electron beam evaporation [27]. A high-temperature thermal treatment above 1,000°C is generally required in order to produce crystallites. All these techniques allow one to form Si-nCs with sizes mainly ranging from 2 to 6 nm, and it is possible to obtain Si-nCs with sizes less than 2 nm in SRO films as deposited with Ro = 30 prepared using the LPCVD technique. Their electronic and optical properties depend on the preparation conditions and methods of fabrication. However, there are some common typical properties for Si-nCs which are independent of the manufacturing technique used. In particular, the nanocrystals' surroundings, which can be either vacuum or some host material like SRO, represent a high potential barrier for carriers (electrons or holes). Such a barrier is often referred to as a confining quantum potential that mainly defines the energy spectrum of the Si-nCs.

There is a large uncertainty in the calculated values of the optical gaps as a function of Si-nC diameter. We can mention two factors influencing the accuracy of the optical gap measurements as follows: First, the nanocrystals which are studied by different research groups have been prepared using different techniques. This fact leads to nanocrystals having different surroundings, surface bonds, and shapes, all of which could lead to scatter in the experimental data. Second, it is difficult to determine exactly the dot sizes and the size distribution in luminescent agglomerate of nanocrystals. Theoretically, the problem persists mainly due to the difficulty to define an appropriate parameter for determining the diameter. For simplicity, a spherical geometry is used in most of the models suggested, since the actual shape of the agglomerates formed and considered as a molecule is totally irregular.

As has been reported in the literature, we can employ a space-filling model, for visualizing the effective shape and relative dimensions of the molecule, in particular, the region of space occupied by it. This model also known as CPK one, is a type of three-dimensional molecular model where the atoms are represented by spheres whose radii are proportional to the radii of the atoms and whose center-to-center distances are proportional to the ones between the atomic nuclei, all in the same scale. We can correlate CPK area and CPK volume, obtaining the diameter *D* (Å) through the equation [9]:

6×CPKvolumeCPKarea=6×43πD234πD22=D

The motivation of this work is to present a theoretical study of the optical and electronics properties of Si-nCs which are embedded in SRO structures regardless of the technique used to fabricate such structures. For this, we review firstly some important experimental results about the measurements of the structural and optical properties carried out on SRO samples grown by different techniques. The aim of this review is twofold; on the one hand, we show relevant information in relationship to actual quantification of Si-nCs about their size, electromagnetic range of emission, molecular structure, and important parameters which are responsible for making variations of these properties. On the other hand, we take this experimental information as background in order to focus correctly our theoretical research predicted by using the DFT method corresponding to atomic composition of different silicon isomers suggested simulating the Si-nC embedded in SRO films.

This paper is outlined as follows: the ‘Methods’ section includes the elemental analytical expressions of energy in nanocrystals. In the ‘Results and discussion’ section, we present two parts: in the first one, we show and discuss the experimental identification and quantification of silicon nanocrystals in SRO films, and then we present and discuss the theoretical results about the structural and optical properties of silicon clusters using DFT method. Finally, the last section presents the conclusions.

## Methods

### Elemental analytical expressions of energy in nanocrystals

Different proposals of elemental models to get analytical expressions of energy of Si-nC are found in the literature, which pretend to explain correctly the energy spectrum of this type of nanostructures. However, this task is not easy because of the complexity associated to these nanoclusters which do not have a well-defined geometry and their composition is nonhomogeneous. Among the several geometries suggested for studying the optical properties of Si-nCs, we find that the spherical geometry is predominantly accepted as a first approximation to understand, to a certain degree, the emission mechanisms in the nanocrystals. In order to obtain the electronic states in a nanocrystal, with a spherical shape, its Hamiltonian, $H=-\frac{\hbar^{2}}{2m}\nabla^{2}+U\left(r\right)$, must be solved. Gaponenko [28] used spherical coordinates *r*, *θ*, and *φ* to solve this Hamiltonian, where the total potential energy *U*(*r*) of the electron inside the spherical region has radial symmetry. By standard methods, the eigenfunctions of this Hamiltonian are found to be $\Psi_{n,l,m}\left(r,\theta ,\varphi \right)=\frac{u_{n.l}\left(r\right)}{r}Y_{\mathrm{lm}}\left(\theta ,\varphi \right)$ where *n* is the principal quantum number, *l* is the orbital number, and *m* is the magnetic number. Here, *Y*_*l*,*m*_(*θ*, *φ*) are the spherical functions. With these solutions *Ψ*_*n*,*l*,*m*_(*r*, *θ*, *φ*) when introduced in the Hamiltonian, we arrive to the equation $-\frac{\hbar^{2}}{2m}\frac{d^{2}u}{dr^{2}}+\left[U\left(r\right)+\frac{\hbar^{2}}{2mr^{2}}l\left(l+1\right)\right]u=\mathrm{Eu}$. The energy values of the electronic states in a spherical nanocrystal are obtained as a solution to this equation, when the potential *U*(*r*) is considered as infinitely high. The energy spectrum is given by $E_{\mathrm{nl}}=\frac{\hbar^{2}\chi_{\mathrm{nl}}^{2}}{2ma^{2}}$, where *a* = 0.543 nm is the lattice constant of Si and *χ*_*nl*_ are the roots of the spherical Bessel functions.

On the other hand, inside the Si-nCs, the electrons and holes are interacting through Coulomb attraction; this fact leads to the formation of excitons. An exciton is known as a bound pair formed by an electron and a hole interacting by Coulomb force forming a hydrogen-like system. When the Bohr radius of the exciton is larger than the size of the nanocrystal, it takes place a quantum confinement. Considering that silicon dioxide surrounds the silicon nanocrystals, and due to it forms a high potential barrier (approximately 9 eV), the excitons are confined within the volume of the nanocrystal. This causes drastic changes in both the electronic band structure and the emission spectrum.

Considering the presence of excitons in the medium and the effects of polarization due to the dielectric oxide surrounding the Si-nC, it can be shown that the Hamiltonian in the effective mass approximation is given by $H=-\frac{e^{2}}{\epsilon \left|r_{e}-r_{h}\right|}-\frac{\hbar^{2}}{2}\left[\frac{1}{m_{e}}\nabla_{e}^{2}+\frac{1}{m_{h}}\nabla_{h}^{2}\right]+\mathrm{polarization}\;\mathrm{terms}$, where *ϵ* is the dielectric function of the nanocluster, and *m*_e_ and *m*_h_ are the effective masses of electron and hole, respectively. Owing to the dielectric function which varies in the cluster by virtue of the nonhomogeneous composition in the surrounding medium, it gives rise to an image charge effect on the electron, i.e., polarization effects which must be added to its total energy as shown qualitative in the last term of *H*. More precisely, the polarization terms refer to the effect of image charge on carriers due to the difference in dielectric constants of the sphere and the surrounding medium. The contribution of polarization terms to the original potential energy *U*_0_(*r*), by and large, is too complex. So, the total potential energy now becomes *U*(*r*) = *U*_0_(*r*) + *V*_sp_(*r*) where *V*_sp_(*r*) is often referred to as a self-polarization term. This term describes an interaction between the electron and its image charge, emerging due to the charge polarization on the boundary between the Si-nC and its dielectric surrounding. It can be represented as [10]$V_{\mathrm{sp}}\left(r\right)=\frac{e^{2}\left(\epsilon_{s}-\epsilon_{p}\right)}{2\epsilon_{s}R}\sum_{l=0}^{\infty }\frac{l+1}{l\epsilon_{s}+\left(l+1\right)\epsilon_{d}}\frac{r^{2l}}{R^{2l}}$, where *ϵ*_s_ and *ϵ*_d_ are the static dielectric constants of bulk silicon and the dielectric surrounding matrix, respectively. It is found that an analytical approximated expression for the lowest eigenvalue of the Hamiltonian including the total energy potential (i.e., the first excited electronic state) is [29]

(1) $$E≅E_{G}-\frac{1.8e^{2}}{\mathrm{\epsilon R}}+\frac{\hbar^{2}\pi^{2}}{2R^{2}}\left[\frac{m_{h}+m_{e}}{m_{e}m_{h}}\right]\text{.}$$

where *E*_G_ is the bulk band gap and *R* is the size of the Si-nC. The first term proportional to *R*^−1^ is the Coulomb term, and the second one proportional to *R*^−2^ is the shift as a result of quantum localization of electrons and holes (quantum confinement). Equation 1 is possible to establish because the correlation between electron and hole positions, induced by the Coulomb interaction, is not enough strong. Independently of confinement energies for electrons and holes, the major effect is additive.

The first theoretical calculation for semiconductor nanoparticle energy is based on ‘effective mass approximation’ (EMA), which was proposed in 1984 by Brus [30]. This approximation allows us to obtain the exciton energy when it is confined to a spherical volume of the crystallite in terms of electron and hole effective masses, and it can be expressed as

(2) $$E=E_{G}+\frac{\hbar^{2}\pi^{2}}{8R^{2}}\left[\frac{m_{h}+m_{e}}{m_{e}m_{h}}\right]-\frac{1.786e^{2}}{4\pi \epsilon_{0}\epsilon_{r}R^{2}}$$

*ϵ*_0_ is the permittivity of vacuum and *ϵ*_r_ is the relative permittivity of Si-nC. Four years later, in 1988 Kayanuma [31] accounted for the electron-hole spatial correlation effect and modified the Brus equation, including a negative term proportional to Rydberg energy.

For very small clusters, there is a large difference between the electron effective mass and the hole one which is much heavier. This fact yields that (*m*_h_ + *m*_e_)/*m*_e_*m*_h_ ≈ 1/*m*_e_. On the other hand, for very small nano-agglomerates, it is found that

(3) $$\mathrm{\Delta E}\approx \frac{h^{2}\pi^{2}}{2m_{e}R^{2}}$$

where Δ*E* = *E* − *E*_*G*_. Apart from this model, many others have been developed with several refinements. The EMA is a rough approximation which assumes the cluster (or nano-agglomerated) to be in a well of infinite potential energy where the Coulomb terms are excluded from the analysis. Brus [32] improved the original model including the Coulomb terms taking into account the effect of the dielectric constant of the matrix on the exciton binding energy and using finite potential wells for calculating energy states. Other EMA models were developed by Kayanuma [31] and Kayanuma and Momiji [33] using quantum confinement finite potentials for modeling clusters with cylindrical and spherical geometries.

The extension of Efros and Efros [22] model suggested by Brus [32] and Kayanuma [31] to include Coulombic energies and correlation effects allows us to derive an expression that models the energies and provides a reasonable guide to the cluster size as a function of *E*_*G*_, in such case the energy is

(4) $$\begin{array}{l} E\left(R\right)=E_{G}+\frac{h^{2}\pi^{2}}{2R^{2}}\left[\frac{m_{h}+m_{e}}{m_{e}m_{h}}\right]-\frac{1.786e^{2}}{\epsilon_{r}R^{2}}\\ \;\;+0.285E_{R} \end{array}$$

where *E*_R_ is the Rydberg energy for the semiconductor in bulk and is given by [34]$E_{R}=\frac{13.606m_{0}}{\epsilon_{r}^{2}\left(\left(m_{e}+m_{h}\right)/m_{e}m_{h}\right)}\;\mathrm{eV}$. In Equation 4, the term *e*^2^ is the Coulombic term and 0.248 *E*_R_ gives the spatial correlation energy which is a minor correction. This method is characterized by the overestimate energy values *E*(*R*), particularly for smaller nano-sized agglomerates with dimensions less than 20 Å. More accurate models using finite barriers give the ratio of the energy gap reduced to the size of the cluster as *R α* 1/*γ* where *γ* is an empirical derivative value in the range from 1.3 to 1.8.

At first glance, it may be redundant to present the above theory related with analytical expressions of energy of Si-nCs, but we must keep in mind that these formulae have frequently used to estimate emission radiation in Si-nC. Nevertheless, their predictions are limited since the theoretical models ignore a lot details that in practice the Si-nCs present themselves. It opens the opportunity to establish a new formalism capable of explaining in depth the underlying physical mechanisms involved in the radiative emission of Si-nCs.

## Results and discussion

### Experimental identification and quantification of silicon nanocrystals in SRO films

#### Identification of vibrational modes in Si-nCs by Raman spectroscopy

**Figure 1:**
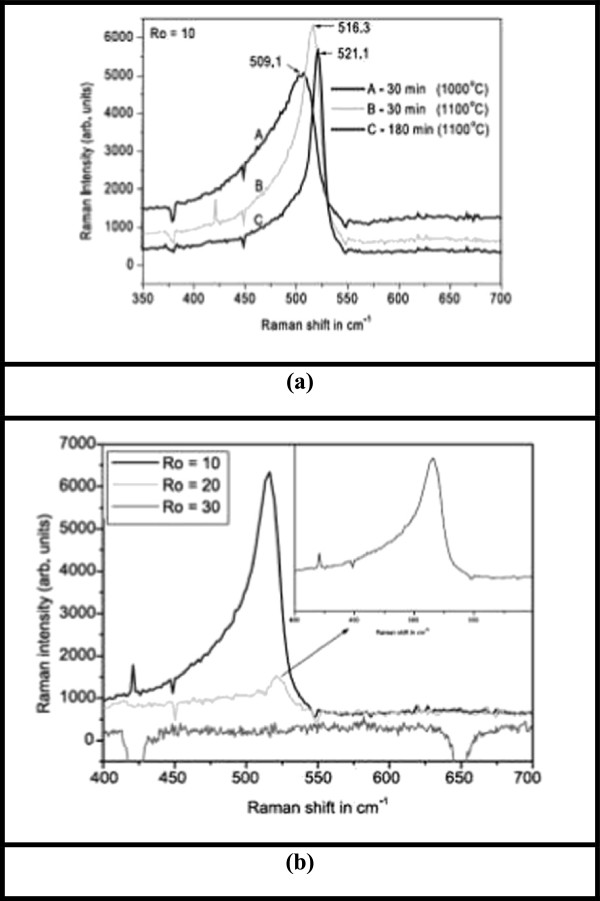
**Backscattering Raman spectra. (a)** Ro = 10 sample, in the region from 350 to 700 cm^−1^ after different annealing times. **(b)** Ro = 10, 20, and 30 after 30 min of annealing. The inset is a zoom of the Raman spectra for Ro = 20.

Raman's effect takes place due to the interaction of optical and vibrational oscillations and results in a variation in vibrational energy of molecule. A phonon is created (anti-Stokes process) or annihilated (Stokes process) during the Raman scattering. Raman spectroscopy as a fast and nondestructive method is recurrently used to characterize the Si nanocrystals. Starting from the shape and the peak position of the first-order Raman scattering band, we can determinate the composition of the nanocrystal, do the estimation of the size of the nanocrystals, corroborate the evolution of the stress on nanocrystals, and estimate the amorphous to crystalline ratio (phase changes). Figure 1 displays the experimental results obtained for SRO thin films deposited by LPCVD technique. Amorphous silicon (a-Si) as well as c-Si can be identified by Raman spectroscopy [35]. A broadband around 480 cm^−1^ is typically associated to a-Si, while bulk silicon has a sharp peak around 521 cm^−1^. For Si-nCs, their peak shifts to smaller wavenumbers, as a function of decreasing size; it has been extensively associated to quantum confinement effects. For pre-annealed films with Ro = 20 and 30, no Raman peaks for Si nanocrystals were observed, but for Ro = 20, a broadband at 485 cm^−1^ indicates the presence of a-Si.

Fascinatingly, the sample with Ro = 10 displays a strong Raman peak for Si nanocrystals at 509.1 cm^−1^ (Figure 1a, the lower curve), indicating that for films with a high Si excess during the 30 min of pre-annealing Si nanocrystals were already formed. After 30 min of annealing at 1,100°C (Figure 1b), a small Raman peak around 521.1 cm^−1^ for Ro = 20 is observed (the middle curve and see inset on Figure 1b), while for Ro = 30 (the lower curve), no peaks were found. Further heating treatment for another 150 min proceeds with this trend; for Ro = 20, an increase of the intensity is observed, whereas for Ro = 30, no characteristic peaks for Si phases were found.

#### Structural characterization of Si-nCs by X-ray diffraction

**Figure 2:**
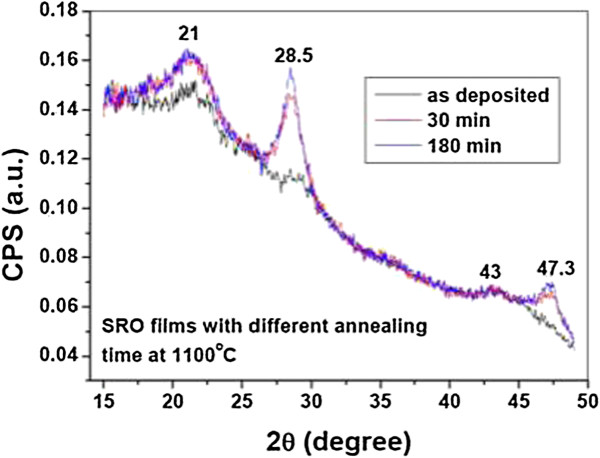
**XRD for SRO**_**10 **_**film deposited by LPCVD with two different annealing times.**

The theory and mathematical representation of XRD by a simple lattice was studied in detail by Laue [36] and Bragg [29], and it is very known that XRD lines of the stressed materials exhibit asymmetrical and broadened line profiles depending on the magnitude of the stress [37]. Therefore, misinterpretation may arise from the fact that both stress and the decrease in coherence length can cause broadening. The method of integral breadths, described by Santra et al. [38] and Warren and Averbach [39] can be used to calculate the effect of both finite grain size and lattice distortion simultaneously. Both methods require the precise measurements of several diffraction lines. In the case of Si nanocrystals embedded in dielectric matrices, Si(111), (220), and (311) peaks are usually observed. However, the signal to the Bragg peaks of Si (220) and (311) sometimes is weak to perform these methods accurately. Figure 2 displays XRD results obtained for SRO_10_ film (an SRO film with Ro = 10) deposited by LPCVD technique. The average size found was from 4.8 to 5.0 nm after 30 and 180 min (annealing times). The broad peak in 21° corresponds to a-SiO_2_, whereas peaks in 28.4° and 47.3° are due to Si (111) and (220), respectively. Incipient peak in 43° could be Si (311).

#### Detection of Si-nC by energy-filtered transmission electron microscopy

By using EFTEM, spatial resolution down to 1 nm can be succeeded. However, EFTEM has no limitations related to the crystallinity of the Si-nCs and can detect both amorphous and crystalline nanoclusters. Moreover, high energy resolution of EFTEM can make it possible to distinguish Si and SiO_2_ plasmon energies.

The raw images obtained from EFTEM measurements were slightly contrasted, and the subtraction of the contribution from SiO_2_ background by yielding a well-defined Si nanocluster was applied. EFTEM is advantageous when analyzing a number of specimens in a short time. Thus, EFTEM is more appropriate for statistical analysis.

**Figure 3:**
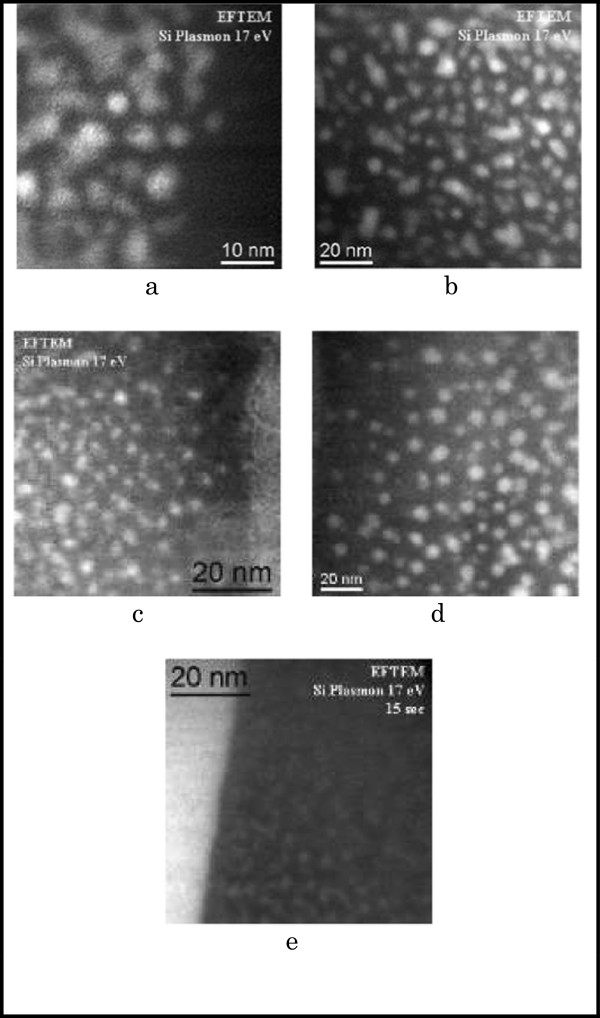
**EFTEM images obtained from SRO thin films.** Focusing silicon agglomerates, under different thermal treatment temperatures and times and varying the relationships Ro. **(a)** Ro = 10, *T* = 1,100°C, 60 min (scale 10 nm), **(b)** Ro = 10, *T* = 1,100°C, 60 min (scale 20 nm), **(c)** Ro = 20, *T =* 1,100°C, 60 min (scale 20 nm), **(d)** Ro = 20, *T* = 1,250°C, 60 min (scale 20 nm), and **(e)** Ro = 30, *T* = 1,100°C, 60 min (scale 20 nm).

Figure 3 displays images obtained for SRO films deposited by LPCVD with different concentrations and annealing times. Figure 3a,b corresponds to Ro = 10 after thermal treatment during 1 h, in two scales; Figure 3c,d corresponds to Ro = 20, with treatment of 1 h, considering two different temperatures; and finally, Figure 3e corresponds to the image of SRO_30_, after annealing at 1,100°C during 1 h.

#### Dimensional estimation of Si-nCs by high-resolution transmission electron microscopy

After Voyles [40] first discerned the distribution of dopant atoms using scanning transmission electron microscopy, active studies are being carried out to examine the single atom or point defects in the crystal lattices. Not many studies, however, were done to investigate point defects in normal HRTEM. It is well known that groups of point defects, either lined up along specific crystallographic orientation or clusters can give rise to contrast in conventional TEM.

**Figure 4:**
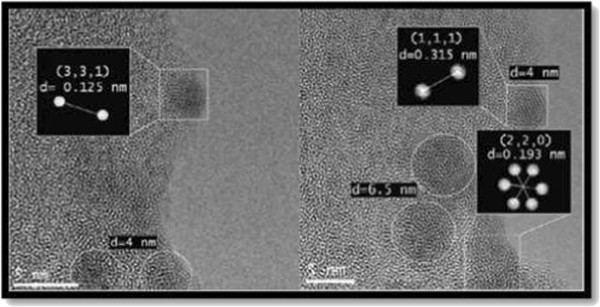
**HRTEM images of the SiO**_***x ***_**films deposited at a growth temperature of 1,150°C.**

We applied HRTEM to obtain images of SiO_*x*_ thin films grown at 1,150°C, and they are shown in Figure 4; HRTEM clearly shows the presence of Si-nCs embedded in SiO_*x*_ films with a size of 4.0 and 6.5 nm. In HRTEM images of thin silicon specimens, bright dots at Scherrer conditions correspond to the tunnels in the structure. In thicker regions, the bright dots in the images correspond to the atomic columns. To distinguish between these two cases in a perfect crystal results to be impossible based only on the image contrast [41].

Figure 4 displays HRTEM images of SiO_*x*_ deposited at 1,150°C by HFCVD technique [42]. On the left side, we obtained agglomerates with a size of 4.0 nm on the average; likewise, on right side, the size was 6.5 nm.

**Figure 5:**
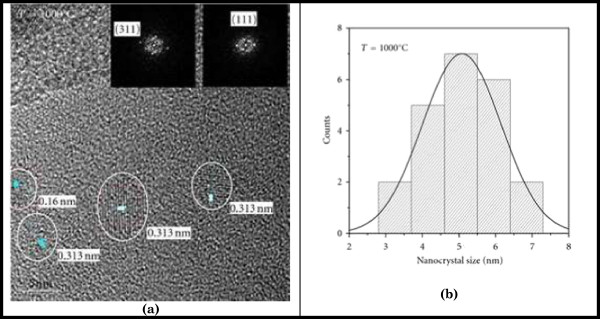
**HRTEM images and nanocrystal size. (a)** HRTEM images of the samples of silicon nanocrystals embedded in SiO_*x*_ matrix obtained by HWCVD grown at 1,000°C. **(b)** Nanocrystal size distribution for samples grown at 1,000°C.

Figure 5a shows the HRTEM images of an SRO thin film deposited at 1,000°C using the hot wire chemical vapor deposition (HWCVD) technique. The insets display Si (311) and Si (111). Average size is displayed in Figure 5b and corresponds to 5.0 nm [43].

#### Chemical bonds in Si-nCs detected by Fourier transform infrared spectroscopy

FTIR is a powerful tool for recognizing types of chemical bonds in a molecule by generating an infrared absorption spectrum that is like a molecular ‘pattern.’

**Figure 6:**
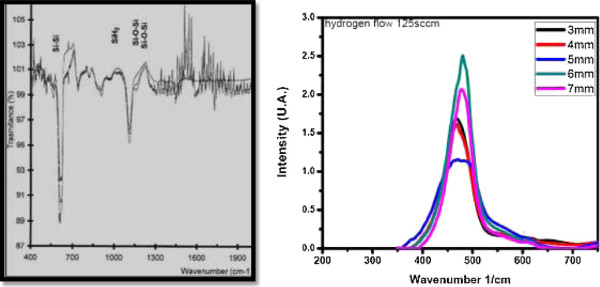
**FTIR spectra.** FTIR absorption spectra from porous silicon layers (left). FTIR spectra of silicon rich oxide sample obtained by HWCVD technique (right).

In Figure 6 (left), we can identify the vibrational frequencies for Si-Si, Si-H, and Si-O-Si bonds found in a sample of porous silicon. Pai et al. [44] have studied the local atomic structure of silicon suboxide (SiO_*x*_, *x* < 2) thin films using infrared (IR) spectroscopy. The films were prepared by plasma-enhanced chemical vapor deposition (PECVD) of silane (SiH_4_) and nitrous oxide (N_2_O) mixtures, which were then diluted with helium. The IR spectra were found to vary significantly with the degree of helium dilution. The films grown without He show SiN, NH, and SiH bonding groups in addition to the three characteristic vibrations of the Si-O-Si linkage. The addition of He reduced the strength of the SiN, NH, and SiH absorption bands and resulted in systematic increases in the frequency of the Si-O-Si asymmetric stretching vibration. The frequency of these Si-O-Si stretching vibrations scales linearly with the oxygen concentration from approximately 940 cm^−1^ in oxygen-doped a-Si to 1,075 cm^−1^ in stoichiometric noncrystalline SiO_2_.

A deposition temperature of 350°C and a He dilution of 50% gave a film composition close to SiO_1.9_. Figure 6 (right) displays the FTIR spectra for a SRO sample obtained by HWCVD using a hydrogen flow of 125 sccm varying the distance from source to substrate from 3 to 7 mm. This sample was annealed at 1,100°C during 1 h [45]. The vibrational frequencies corresponding to the five peaks are as follows: 467.2, 471.3, 475.5, 478.3, and 481.2 cm^−1^.

#### Radiative emission in Si-nCs measured by photoluminescence technique

**Figure 7:**
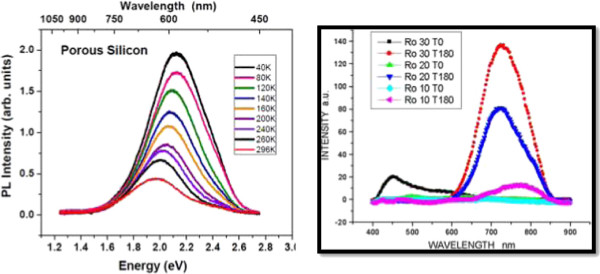
**PL spectra and photoluminescence.** PL spectra obtained from a sample of porous silicon at different temperatures, measured in vacuum conditions (left side) and photoluminescence for SRO with Ro = 10, 20, and 30 before (T0) and after (T180) annealing at 1,100°C for 180 min, for an excitation wavelength of 270 nm (right side).

Figure 7 (left) shows the photoluminescence spectra of a sample of porous silicon at different temperatures; the excitation line was 407 nm (60 mW), and the measurements were performed in vacuum. It can be seen that each peak is symmetric, which means a good surface morphology and a material without defects, with a shift towards higher energy inversely with temperature, with a ratio of 5.85E − 4 eV/K. The peaks of the spectra are contained approximately between 1.65 and 2.6 eV (475 to 750 nm). The highest intensity peaks fit well with temperature by the relation *E* (eV) = 2.1636 − 0.000585 *T* (K), for 40 ≤ *T* ≤ 300 K. These spectra are shown because the origin of emission in p-Si is not well-known, and the Si-nCs generated in this material may contribute to explain luminescence measured experimentally.In Figure 7 (right), we display the PL emission spectra observed for the SRO films, when they are deposited by LPCVD technique. Clearly, the emission of SRO varies in the range from 400- and 850-nm wavelengths. Experimentally, our researching group has not observed emission outside of this range, not even with the high excitation energy of cathode electrons.

As can be seen in Figure 7 (right), photoluminescence is only observed in annealed samples [46] (curves labeled with T180 mean, 180 min of annealing time at 1,100°C). As a matter of fact, only samples annealed at 1,100°C produce high emission, and the highest photoemission is obtained for SRO30 (Ro = 30) films. The PL is only obtained from the visible (VIS) to the near-infrared (NIR) range, and its intensity reduces with Ro decreasing. Comparatively, SRO10 produces negligible emission as that of SRO30. Also, we can appreciate a wavelength shift to blue emission when Ro is increased (higher levels of energy)

### Theoretical results about the structural and optical properties of silicon clusters using DFT method

In 1980, Pulay published a method [47] known as the direct inversion of the iterative subspace (DIIS). Like the Davidson method [48], DIIS applies direct methods to a small linear algebra problem (now a system of linear equations instead of an eigenvalue problem) in a subspace formed by taking a set of trial vectors from the full-dimensional space. Pulay found that DIIS could be useful for accelerating the convergence of self-consistent field (SCF) procedures and, to a lesser extent, geometry optimizations. In a previous paper [9], we employed a SCF model with a restricted hybrid (Hartree-Fock/density functional theory) HF-DFT-SCF calculation using Pulay mixing + geometric direct minimization level of theory, compiled in the SPARTAN 08/10 software package [41] for evaluating small silicon clusters.

Møller-Plesset (MP) perturbation theory is one of the several quantum chemistry post-Hartree-Fock *ab initio* methods in the field of computational chemistry. It improves on the Hartree-Fock method by adding electron correlation effects by means of the Rayleigh-Schrödinger perturbation theory (RS-PT), usually to the second (MP2), third (MP3), or fourth (MP4) order. Their main idea was published as early as 1934 by Møller and Plesset [49]. The MP2/6-31G (d) level of theory is selected for geometry relaxation to approximately account for the correlation effect of all electrons to the geometric structures. In order to identify the most stable isomers among nearly degenerated isomers, Zhu and Zeng [50] performed calculations for single point of the coupled cluster single or double substitution from the Hartree-Fock determinant CCSD (T)/6-31G (d), where (T) refers to include triple excitations non-iteratively [51].

The CCSD (T) method is often called the ‘gold standard’ of computational chemistry [52], because it is one of the most accurate methods applicable to reasonably large molecules. It is particularly useful for the description of non-covalent interactions where the inclusion of triple excitations is necessary for achieving a satisfactory accuracy. While it is widely used as a benchmark, the accuracy of CCSD (T) interaction energies has not been reliably quantified yet against more accurate calculations.

Moreover, for the isomer with the lowest CCSD (T)/6-31G (d) energy, its stability was further examined by calculating its vibrational frequencies at the HF/6-31G (d) level of theory (a hybrid density functional model using a medium-sized basis set) [53]. Structures of the low-lying isomers of Si_15_-Si_20_ have been reported in the literature [17,24], and some of them are likely true global minima.

#### Structural and optical properties calculated for isomers Si_15_

Since 1998, Ho et al. [54] employed the unbiased tight-binding model (TBM) search and disclosed the low-lying clusters 15A-15D which contain the capped trigonal prism unit form. The isomer having the global minimum at the CCSD (T)/6-31G (d) level is the 15A (C_3v_) one whose geometry is a tri-capped trigonal prism fused with a tri-capped trigonal anti-prism. The vibrational frequency analysis at the MP2/6-31G (d) level shows that the 15A isomer has two imaginary frequencies. Thus, isomer 15A (C_3v_) may not be a stable structure but a transition-state structure at the MP2/6-31G (d) level of theory. A structural perturbation to 15A (C_3v_) followed by geometry relaxation gives rise to isomer 15A with Cs symmetry (possesses only a mirror plane *σ*_h_). Isomer 15A (Cs) shows no imaginary frequencies. Ours results confirm that isomer 15A has no symmetry (C_1_) and is the global minimum.

**Figure 8:**
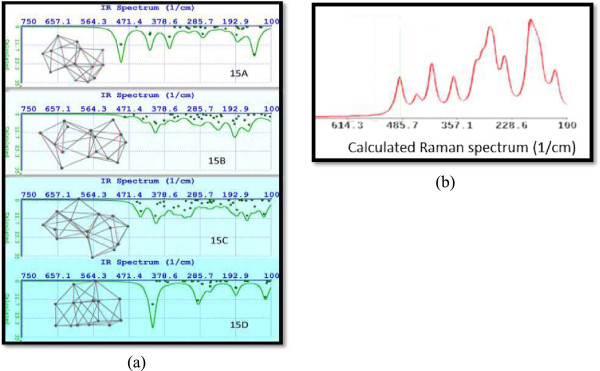
**Molecular structure and FTIR spectra calculated for isomers Si**_**15 **_**(a) and calculated Raman spectrum for Si15A (b).**

Figure 8a displays the molecular structures and FTIR spectra for Si_15_ isomers. Isomers 15B and 15C show the less intensity with a scattered distribution and not well-defined peaks. There are 39 vibrational modes in these isomers.

On the other hand, isomers 15A and 15D have a variety of defined peaks. The most intense peak for isomer with the global minimum (15A) corresponds to a frequency of 493.086 cm^−1^, and the local minimum with the highest difference in energy (15D) displays the most intense peak at 409.9 cm^−1^.

**Figure 9:**
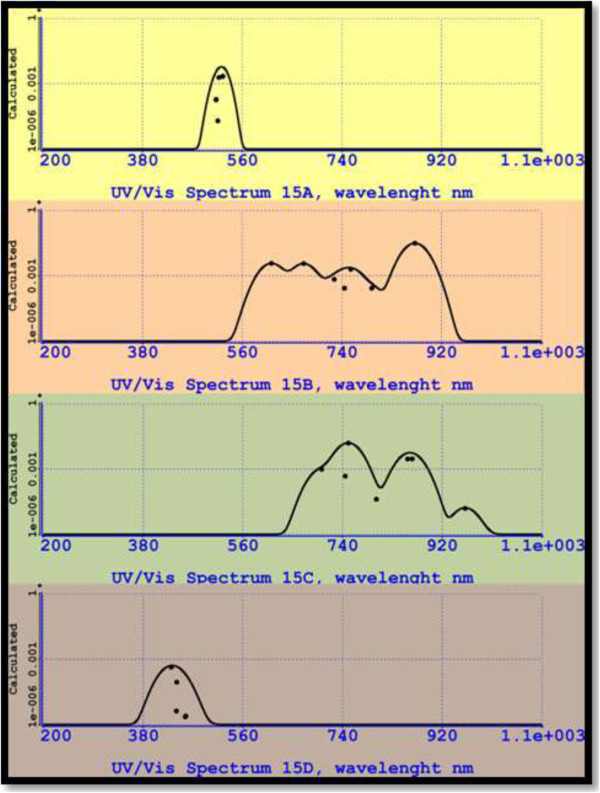
UV-VIS spectra for silicon isomers from 15A to 15D.

We have calculated Raman spectra for all previously cited Si_15_ isomers. Figure 8b corresponds to calculated Raman spectrum for isomer Si15A. Several models have been proposed for size calculations using Raman line shape. The model suggested by Richter, Wang, and Ley [55] (RWL) and later improved by Islam and Kumar [56] and by Mishra and Jain [57] has widely been employed. Regrettably, RWL model stands on the multiplication of the wave function in an infinite crystal by an arbitrary weighting function *W*_*D*_(*r*) (Gaussian or sine functions) in order to find the dimensions of the silicon clusters. This undiscriminating choice results in quite different values at low dimension of nanocrystals [58,59].In Figure 9, we display the UV-VIS spectra for silicon isomers from 15A to 15D. We can appreciate remarkable differences in these spectra. So, for example, the isomer 15A results with emission located at a narrow intense peak at 525.31 nm (green emission) and the silicon isomer 15D with a wider peak at 440.88 (blue emission).

Results obtained for isomers 15B and 15C predict emission in most of the visible region and extends until the IR region. For isomer 15B, the most intense peak lies in IR and has a wavelength of 871.74 nm, whereas for isomer 15C, the most intense peak lies at 750.42 nm (red emission).

Table 1 contains numerical data of the geometric and optical properties for isomers ranging from Si_15_ to Si_20_. For isomers Si_15_, the differences in the values of the energies obtained with respect to isomer 15A confirm undoubtedly the global minimum.

**Table 1:** **Structural and optical properties calculated for isomers varying from Si**_**15 **_**to Si**_**20**_

**Isomer**	***E *** **(au)**	**rel. *E *** **(eV)**	**Band gap (eV)**	**Dipole (Debye)**	***D *** **(nm)**	**Polarizability/atom**	**ZPE (eV)**	**Ovality**	**UV-VIS lambda max**
15A	−4,342.34404	0.00	3.051	3.097	0.72508	4.629	0.632	1.2115	525.314
15B	−4,342.32497	0.52	1.889	0.959	0.72991	4.648	0.629	1.2035	871.635
15C	−4,342.31698	0.74	1.971	0.525	0.72724	4.656	0.614	1.2099	750.415
15D	−4,342.30761	0.99	3.441	0.027	0.75043	4.594	0.589	1.1645	431.109
16A	−4,631.80268	0.18	1.954	0.00	0.75701	4.465	0.636	1.1833	750.886
16B	−4,631.80953	0.00	2.450	0.54	0.73474	4.456	0.663	1.2189	583.651
16C	−4,631.79720	0.33	2.250	0.76	0.75324	4.469	0.625	1.1911	630.165
17A	−4,921.27172	0.8	1.053	2.384	0.70364	4.356	0.706	1.2540	1,033.850
17B	−4,920.97362	8.659^a^	0.619	0.180	0.76368	4.494	0.886	1.4320	2,747.970
17C	−4,921.30164	0.00	1.874	0.638	0.75456	4.311	0.757	1.2104	838.4698
18A	−5,210.83526	0.00	2.255	2.84	0.74512	4.177	0.80	1.2504	552.447
18B	−5,210.80158	0.92	1.388	0.04	0.73920	4.178	0.74	1.2580	692.813
18C	−5,210.80010	0.96	1.323	0.01	0.73906	4.178	0.73	1.2581	1,732.74
18D	Not converged								
19A	−5,500.25750	1.98	1.878	3.45	0.7361	4.098	0.81	1.2965	700.980
19B	−5,500.33041	0.00	2.198	1.93	0.7544	4.058	0.83	1.2571	643.427
19C	−5,500.28806	1.15	2.351	1.21	0.7259	4.076	0.82	1.3112	678.640
20A	−5,789.84528	0.00	2.7320	1.54	0.74238	3.9476	0.92	1.3005	549.724
20B	−5,789.81937	0.71	2.9045	0.00	0.75361	3.9465	0.87	1.2813	540.264
20C	−5,789.76867	2.08	1.6437	0.73	0.77313	3.9727	0.90	1.2514	756.136

^a^Isomer Si17B presents and unexpected energy.

The band gap calculated for the most stable isomer is 3.051 eV. Also, a great value for dipole moment was obtained (3.097 Debye). The nano-agglomerate size of the global minimum has resulted to be the lowest of this set of isomers (0.72508 nm). Finally, the highest zero-point energy (ZPE) and ovality values correspond to the global minimum.

#### Structural and optical properties calculated for isomers Si_16_

For Si_16_ cluster, Zhu and Zeng [50] found that isomer 16A with C_2h_ symmetry gives the lowest energy at the CCSD (T)/6-31G (d) level, similar to the prediction of Ho et al. [54]; nevertheless, we have found that isomer 16B is the one with the global minima with a difference of only 0.18 eV with respect to isomer 16A.

The isomer 16A can be described as two fused pentagonal prisms. Its structure is unique in the sense that it is neither based on the tri-capped trigonal prism motif (as 16B) nor based on a stacking sequence of fourfold and fivefold rings with capping atoms (as 16C).

Numerical data for isomers 16A to 16C are listed in Table 1. The isomer 16B exhibits not only the lowest energy and the highest band gap (2.45 eV), ovality, and ZPE but also the lowest parameter *D*, polarizability/atom, and the lowest wavelength for the most intense peak, for this set of isomers.

**Figure 10:**
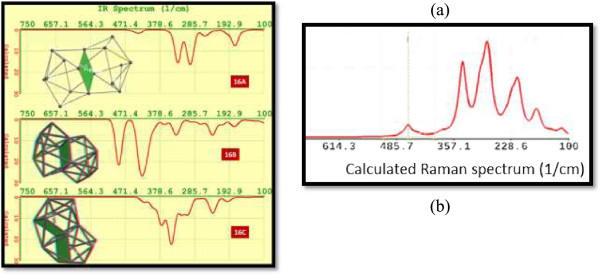
**FTIR spectra and calculated Raman spectrum. (a)** FTIR spectra for isomers from 16A to 16C. The insets on the left side correspond to the molecular structures of these isomers. **(b)** Calculated Raman spectrum for Si16A.

Figure 10a displays the structure and FTIR spectra for isomers Si_16_. For isomer 16A, we can appreciate five peaks, two of them which are the most intense, at the frequencies of 295 and 331 cm^−1^. For isomer 16B, the frequencies of the two most intense peaks correspond to 415 and 487 cm^−1^. In the central position of the atomic structures (left side figures) of these two isomers, we see four silicon atoms forming a cycle. On the other hand, isomer 16C is somewhat different, with only one intense peak in 345 cm^−1^. The 16C atomic structure does not display a cycle in the middle part of the nanocrystal. Instead of that, there are two cycles with four silicon atoms each. In all the cases, we have obtained an imaginary frequency of vibration for isomers Si_16_.

**Figure 11:**
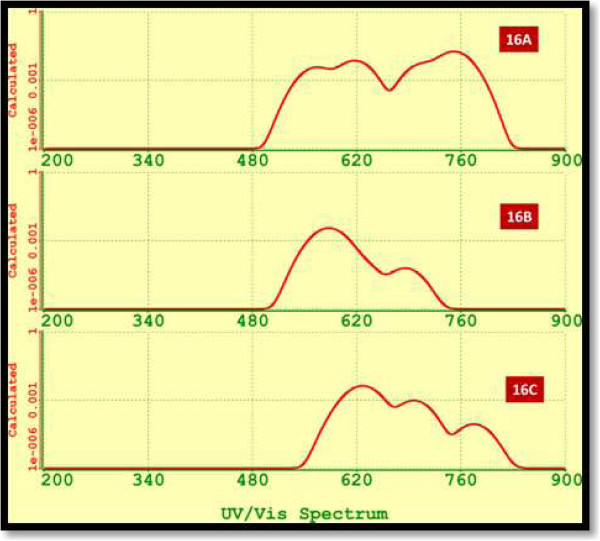
UV-VIS spectra for silicon isomers 16A to 16C.

Raman spectroscopy, which is a sensitive probe to the local atomic arrangements and vibrations, has been used to describe Si nanostructures [60]. The shift and half-width of the one-phonon Raman peak in c-Si have often been used to obtain an estimate of the characteristic dimensions of the Si crystallites. We have evaluated theorically the Raman spectra for all Si_16_ isomers included in this work. Particularly in Figure 10b, we show the calculated Raman spectrum for isomer Si16A.Figure 11 shows the UV-VIS spectra for isomers 16A to 16C. It is noticeable that these silicon nano-agglomerates will emit in the visible region.

For isomer 16B, the highest intensity of emission corresponds to 583.65 nm, and the second peak observed is at a wavelength of 576 nm (both in yellow color). We observe a redshift for the other two isomers. Isomer 16B displays a wideband of emission (from green to red color), with the most intense peak at 750.89 nm (red color), while isomer 16C has the most intense calculated emission at 630.16 nm.

#### Structural and optical properties calculated for isomers Si_17_

Ho et al. [54] have reported for Si_17_ isomers, 17A, with C_3v_ symmetry as ‘possibly the lowest energy structure,’ and it does contain a tri-capped trigonal prism (TTP) unit and a hexagonal chair unit. The six-atom hexagonal chair unit can be viewed as a fragment in bulk diamond silicon [61,62]. The calculated emission in this work confirms this assertion (emission in the near infrared, see Table 1).

It is interesting to note that the more spherical-like Si_17_ isomer, 17C, is very competitive in stability compared to the prolate-shaped isomer 17A [59]. Using a level theory of HF/6-31G*, Zhu and Zeng [50] report isomer 17C as the structure with the global minimum in energy for Si_17_ isomers, and isomer 17A using other levels of theory (MP2/6-31G*, MP3/6-31G*, MP4(SDQ)/6-316*, CCSD/6-31G*, CCSD(T)/6-31G*). Isomer 17B exhibits an unusual energy difference of 8.659 eV with respect to the global minimum (as can be seen in Table 1). During our calculations, it had instability and troubles to get convergence. Probably, this was the reason for which Zhu did not report the evaluation of isomer 17B using other levels of theory.

**Figure 12:**
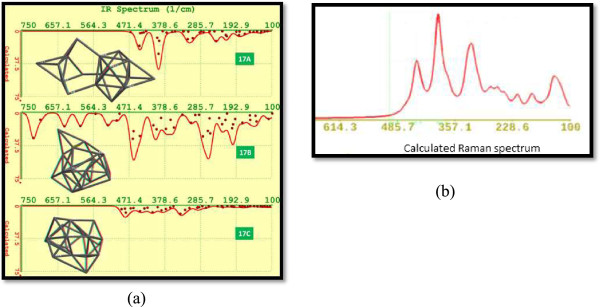
**Calculated FTIR spectra and Raman spectrum. (a)** Calculated FTIR spectra for isomers from 17A to 17C. The structures of these isomers are depicted on the left side. **(b)** Calculated Raman spectrum for Si17A.

The FTIR spectra for isomers Si_17_ are displayed in Figure 12a. Particularly, it attracts attention the fact that the isomer 17B displays calculated vibrational frequencies above 480 cm^−1^. The intensity calculated for vibrational frequencies for isomer 17C was very small with respect to the other two isomers Si_17_.

The Raman shift due to the quantum confinement can be described by a phenomenological approach founded on the negative dispersion of optical phonons with finite momentum and the averaging and folding of phonon frequencies in small particles. An analytic expression of this approach to describe the Raman frequency shift as also employed by Paillard et al. [58] is $\Delta \omega =-52.3{\left(\frac{0.543}{L}\right)}^{1.586}\text{,}$ in which 0.543 nm is the lattice constant of silicon, *L* is the crystallite size, the parameter *A* = 52.3, and the exponent *γ* = 1.586 which are used to explain the vibrational confinement due to the finite size in a nanocrystal, and these values depend on each specific system. We have evaluated all Raman spectra corresponding to Si_17_ isomers A to C, using DFT. Figure 12b shows, as an example, the spectrum which corresponds to isomer Si17A.

**Figure 13:**
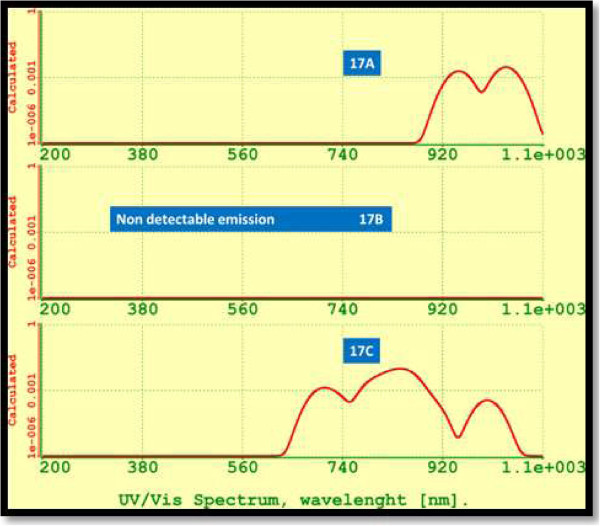
UV-VIS spectra for silicon isomers 17A to 17C.

Figure 13 shows the UV-VIS spectra for isomers Si_17_. The calculated wavelength for the emission of the most stable Si_17_ isomer (17C) corresponds to a wideband from red to NIR (702.69 to 1,000.09 nm) with a maximum calculated intensity of 838.4698 nm.

Calculated emission for isomer 17A results in a narrowband with emission only in the near infrared. The predicted emission for isomer 17B is in the tail infrared but results with a very low intensity.

#### Structural and optical properties calculated for isomers Si_18_

**Figure 14:**
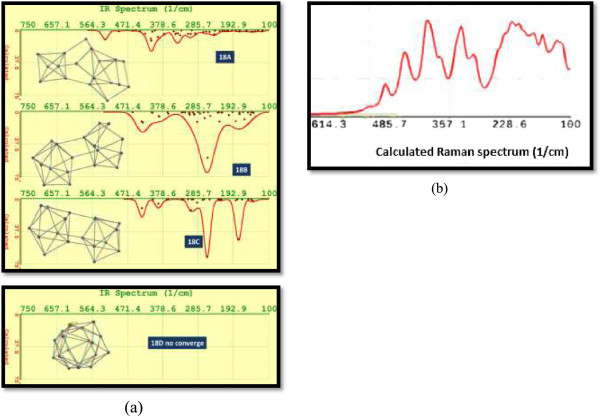
**Calculated FTIR spectra and Raman spectrum. (a)** Calculated FTIR spectra for silicon isomers from 18A to 18C. In a rectangle, at the bottom of this figure, we show the structure for isomer 18D; this isomer did not have convergence, and only the molecular structure is shown. **(b)** Calculated Raman spectrum for Si18A.

With respect to Si_18_ cluster, four low-lying isomers were considered, and they are shown in Figure 14. The elongated isomer 18A has the lowest energy at the HF/6-31G* level of theory. Isomer 18A has the structure similar to the ground-state structure of Si_18_^**+**^, predicted by Ho et al. [17]. It contains a magic number cluster Si_6_ unit and a hexagonal chair unit. A slight structural perturbation to this C_3v_ isomer followed by geometry relaxation gives isomer 18B with Cs symmetry. Both 18B and 18C with C_2v_ symmetry contain a tri-capped trigonal prism unit, and both are very viable in stability compared to 18A because the calculated difference in energy was 0.92 and 0.96 eV, respectively (see Table 1). Isomer 18D is a new isomer with high symmetry but relatively high energy. It is composed of two-capped tetragonal anti-prisms, and in this case, we did not obtain convergence at HF/6-31G* level of theory (see Figure 14a and Table 1).

FTIR spectra for isomer 18A shows the most intense calculated response at the vibrational frequency of 409 cm^−1^; nevertheless, frequencies of 450 and 458 cm^−1^ were calculated too.

The shift of the phonon peak towards lower wavenumbers and broadening of the peak width, in the Raman spectra, are attributed to the confinement of optical phonons in nano-dimensional Si crystals [63]. The shift of the phonon peak could be used to calculate the crystallite size of Si. Using DFT, we have evaluated the Raman spectra for isomers Si_18_ A to C. In Figure 14b, we display the Raman spectrum for Si_18_ A. In this case, the phonon peak appears at a frequency of 485.7 cm^−1^.

The frequencies that have been observed in experimental reports are a little higher than the calculated ones for this isomer (see Figure 6, left). Isomer 18B displays the most intense peak at 260 cm^−1^. Each isomer Si_18_ has 48 vibrational modes. Isomer 18C presents the two most intense peaks at the vibrational frequencies 179 and 262 cm^−1^; and two of the calculated wavenumber were imaginary.

**Figure 15:**
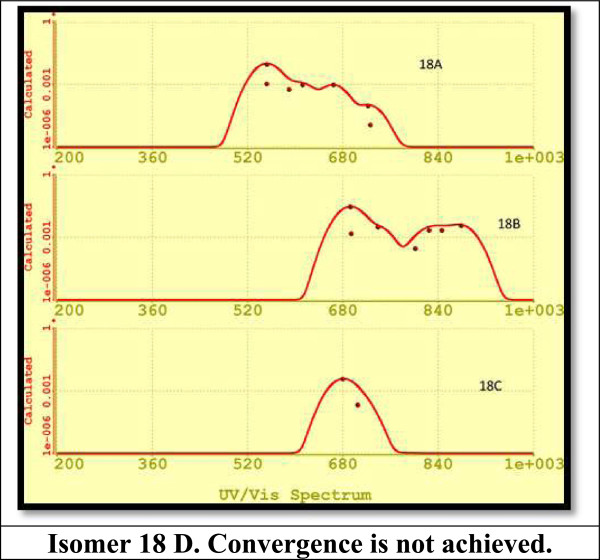
UV-VIS spectra for silicon isomers from 18A to 18C.

Figure 15 displays the UV-VIS calculated spectra for isomers Si_18_. The results obtained for these isomers predict emissions in a part of the visible range, from green to red color, and IR. DFT predicts green emission due to the most intense peak for isomer 18A and in red color for isomer 18B, and the most intense peak for isomer 18C occurs in the IR region at 1,732.74 nm (not shown in Figure 14, refer to Table 1). For isomer 18B, additionally to emission displayed in Figure 14, we obtained a calculated emission wavelength in IR at 1,620.05 nm.

#### Structural and optical properties calculated for isomers Si_19_

Ho et al. [54] found for Si_19_ cluster a spherical-like isomer 19A. The isomer 19B which contains a tetra-capped trigonal prism unit and a magic number cluster Si_6_ unit is very competitive in stability compared with 19A. Isomer 19B was found based on a novel single-parent evolution algorithm coupled with DFTB/DFT methods. Isomer 19C is composed of a TTP unit and a Si_10_ (bi-capped tetrahedral anti-prism) unit [54]. Its energy is slightly higher than both 19A and 19B.

**Figure 16:**
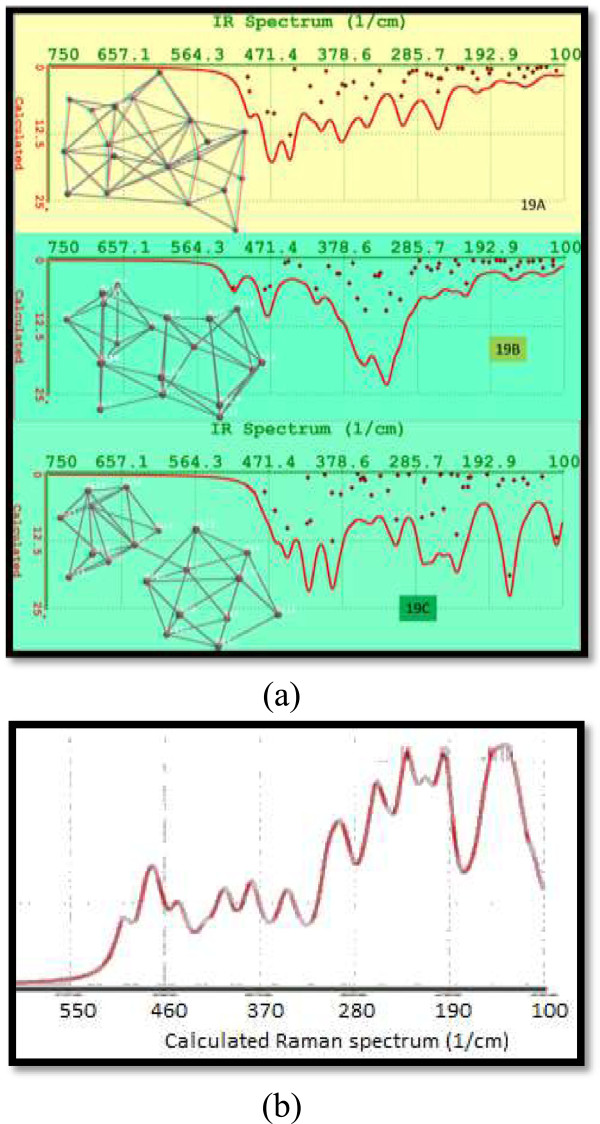
**Calculated FTIR spectra and Raman spectrum. (a)** Calculated FTIR spectra for isomers 19A to 19C. The molecular structures of these isomers are depicted on the left side. **(b)** Calculated Raman spectrum for Si19A.

Molecular structures for three isomers Si_19_ were calculated. The nano-agglomerates Si_19_ are displayed on the left side of Figure 16a. The structure 19B was energetically the most stable, having differences in energy with values of 1.98 and 1.15 eV with respect to silicon agglomerates 19A and 19C. Also, structure 19B has an intermediate value of band gap with respect to analogous isomers.

Figure 16a displays the FTIR spectra for isomers Si_19_ A, B, C. We can appreciate manifold vibrational frequencies varying from 200 to 500 cm^−1^. The strongest vibrations, for the most stable isomer, include the most intense peak at 325 cm^−1^, with an adjacent shoulder around 351 cm^−1^. The highest vibration is due to silicon atom located in the highest part or trigonal prism. The third most important vibration of isomer 19B occurs at 472 and 479 cm^−1^ and is associated to the bending mode Si-Si vibration of atoms in the base of the trigonal prism.

The atomic structure of isomer 19C seems like two small agglomerates joined, along the central part, for a simple silicon-silicon bond. The isomer 19C is the isomer Si_19_ which has the smallest size and dipole moment of all but presents the highest ovality (see numerical data in Table 1).

Raman spectroscopy provides useful information about the structure of silicon nano-agglomerates. The position and intensity of peaks in the spectrum reflect the molecular structure and can be used to determine the chemical identity of the sample. No two structures give exactly the same Raman spectrum, and the intensity of the scattered light is proportional to the amount of material present. Raman spectroscopy has shown to be useful for investigating the physical properties such as crystallinity, phase transitions, and isomers or polymorphs. We evaluate the Raman spectra for Si_19_ agglomerates. Figure 16b displays the Raman spectrum for Si19A.

**Figure 17:**
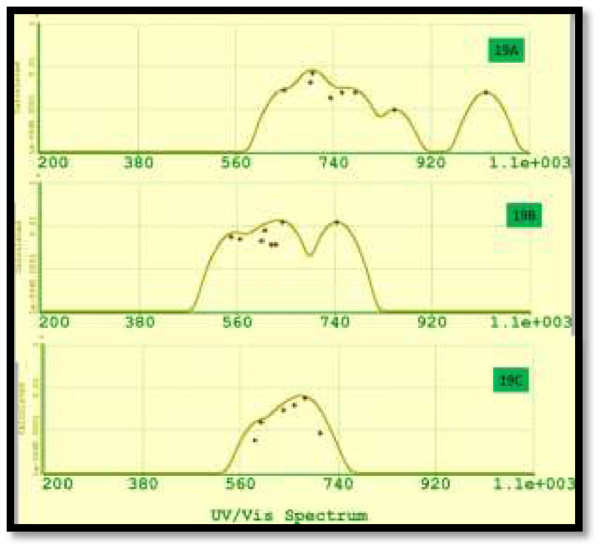
UV-VIS spectra for silicon isomers 19A to 19C.

Finally, in Figure 17, we display the UV-VIS spectrum for Si_19_ isomers. These three isomers resulted with calculated emission in the visible region. Besides, isomer 19A extends its luminescence until the near infrared. Isomer 19B which is more stable energetically has the widest band.

## Conclusions

In this work, we calculated theoretically the IR, UV-VIS, and Raman spectra, the orbital energy levels including the frontier orbitals (HOMO and LUMO) and a selected set of geometric properties of medium-sized silicon agglomerates from Si_15_ to Si_20_ including all stable isomers. These Si-nCs present a size which is less than 1 nm. Their gap energies oscillate between 1.053 and 3.051 eV. The equilibrium energy calculated for several proposed Si clusters at ground state and at the first six excitation states calculated results very useful to evaluate the possible impact to the PL from different silicon structures present in SRO films. From the theoretical PL spectra obtained in this work, we can conclude that this family of silicon agglomerates emits in the visible region, extending in some cases to the near infrared.

## Competing interests

The authors declare that they have no competing interests.

## Authors’ contributions

NDET conducted the modeling of silicon nano-agglomerates and wrote the draft manuscript. DHL provided the background and support in the modeling process and corrected the draft manuscript. JFJFG provided the idea and supervised the study. JALL provided high-resolution transmission electron microscopy (HRTEM) and EFTEM studies and supervised the film growth procedures. JMJ carried out the PL and X-ray measurements in silicon samples. DEVV conducted the SiO_*x*_ film growth. All authors read and approved the final manuscript and contributed to the analysis and discussion of results.

## Authors’ information

NDET is currently a Ph.D. candidate at the Researching Center for Semiconductors Devices (IC-CIDS) in the Science Institute of Autonomous University of Puebla, Mexico. He has been working on the origin of luminescence in silicon and silicon rich oxide thin films. His research interests include modeling using molecular mechanics, semi-empirical and Hartree-Fock methods, and density functional theory, and material science including methods for deposition and characterization techniques of semiconductors and superconductors. He also has explored other topics including phtalocyanines, graphene, and carbon nanotubes. DHL is currently a researcher and professor in the Science Institute - Center of Investigation for Semiconductors Devices (IC-CIDS) of Autonomous University of Puebla, Mexico. He has been working on the optical properties of semiconductors in the framework of local and nonlocal theory, Casimir forces with dispersive spatial effects, and luminescent effects in compound semiconductor. Recently, his research interest is focused on spintronics in semiconductor materials and luminescence in graphene and nanotubes of carbon. JFJFG is currently a researcher and professor in the Science Institute - Center of Investigation for Semiconductors Devices (IC-CIDS) of Autonomous University of Puebla, Mexico. He has been working on silicon rich oxide, specifically in luminescence. Also, he has been working with porous materials (porous silicon and porous aluminum), their structure and electrical and optical properties. His research interests are the conduction process and the modeling of these processes. JALL is currently a researcher and professor in the Science Institute - Center of Investigation for Semiconductors Devices (IC-CIDS) of Autonomous University of Puebla, Mexico. He has been working on the structural, electrical, and optical characterization of materials and MOS structures. His research interest is the physics and technology of nanostructured materials and silicon devices. Additionally, his research interests are, too, the nanotechnology, material characterization, and optoelectronic devices such as sensor, LEDs, and solar cells. JMJ is currently a researcher and professor in the Science Institute - Center of Investigation for Semiconductors Devices (IC-CIDS) of Autonomous University of Puebla, Mexico. He has been working on compound semiconductor families III-V (GaAs, GaSb, GaAlAs, GaAlSb, GaAlSbAs, InGaSbAs), II-VI (ZnO, ZnS, CdO, CdS, CdSe, CdSSe), and IV-VI (PbS, WO_3_, Cu_2_O), epitaxial growth by LPE, synthesis by spray pyrolysis and chemical bath, semiconductor devices (LD and APDs), and structural and photoluminescence characterization of materials and devices. DEVV is currently a Ph.D. student in the Science Institute - Center of Investigation for Semiconductors Devices (IC-CIDS) of Autonomous University of Puebla, Mexico. She started to work on the growth and characterization of nonstoichiometric silicon oxide obtained by HFCVD. Her research interests include experiments and structural, optical, and electrical characterization of SiO_*x*_ films and MOS structures.
